# ACKNOWLEDGING ILLNESS WHEN LIFE IS “CUT SHORT”: INCREASED COMMUNION IN LEGACIES

**DOI:** 10.1093/geroni/igad104.2685

**Published:** 2023-12-21

**Authors:** Mary Kate Koch, Sophia Maggiore, Carma Bylund, Susan Bluck

**Affiliations:** University of Florida, Gainesville, Florida, United States; University of Florida, Gainesville, Florida, United States; University of Florida, Gainesville, Florida, United States; University of Florida, Gainesville, Florida, United States

## Abstract

Crafting a legacy is meaningful activity in later life (McAdams et al., 1993). Threat of dying affects how individuals frame their life legacy (Hunter, 2007). We argue feeling one’s life is being “cut short” may promote greater communion in legacies. Older individuals with cancer may, however, need to acknowledge their illness to create a communion-focused legacy. To assess being ‘cut short, we calculated expected years of life lost (EYLL): difference between participant age and average U.S. life expectancy for men and women (NCHS, 2022). EYLL and self-reported Acknowledgement of Illness were used to predict extent of communion shared in personal legacies. Participants were 204 older adults with cancer receiving outpatient palliative care (M=65.78 years, Range= 55-88 years, 65.69% women) who narrated their legacy during Dignity Therapy (Chochinov, 2005). Narratives were audio-recorded, transcribed and reliably (κ=.71–1) content-analyzed for communion themes (McAdams, 2001). Hierarchical regression analyses (adjusted for gender and cancer stage) examined EYLL, Illness Acknowledgement and their interaction. Interaction between EYLL and illness acknowledgment showed participants with more expected years to lose narrated more total communion but this was particularly true for those acknowledging they were terminally ill, F(7, 173)=2.42, p=.02. Overall, older adults with cancer appear to value communion as an important aspect of legacy. Note, however, that individuals whose life is ‘cut short’ were especially likely to weave communion into their legacy. This may indicate a need to cement their legacy during DT, given they have had less time to do so in daily life or formal documents.

